# Circling the drain: the extinction crisis and the future of humanity

**DOI:** 10.1098/rstb.2021.0378

**Published:** 2022-08-15

**Authors:** Rodolfo Dirzo, Gerardo Ceballos, Paul R. Ehrlich

**Affiliations:** ^1^ Department of Biology, Stanford University, Stanford, CA 94305, USA; ^2^ Instituto de Ecología, Universidad Nacional Autónoma de México, Mexico City 04510, Mexico

**Keywords:** mass extinctions, among-driver synergies, complex adaptive systems, population loss, human enterprise

## Abstract

Humanity has triggered the sixth mass extinction episode since the beginning of the Phanerozoic. The complexity of this extinction crisis is centred on the intersection of two complex adaptive systems: human culture and ecosystem functioning, although the significance of this intersection is not properly appreciated. Human beings are part of biodiversity and elements in a global ecosystem. Civilization, and perhaps even the fate of our species, is utterly dependent on that ecosystem's proper functioning, which society is increasingly degrading. The crisis seems rooted in three factors. First, relatively few people globally are aware of its existence. Second, most people who are, and even many scientists, assume incorrectly that the problem is primarily one of the disappearance of species, when it is the existential threat of myriad population extinctions. Third, while concerned scientists know there are many individual and collective steps that must be taken to slow population extinction rates, some are not willing to advocate the one fundamental, necessary, ‘simple’ cure, that is, reducing the scale of the human enterprise. We argue that compassionate shrinkage of the human population by further encouraging lower birth rates while reducing both inequity and aggregate wasteful consumption—that is, an end to growthmania—will be required.

This article is part of the theme issue ‘Ecological complexity and the biosphere: the next 30 years’.

## 1. A sixth mass extinction: the context

Five major episodes of mass biological extinction (*sensu* Jablonski [1]: those with at least 76% of species lost) have occurred over the last 550 million years (Myr)—that is, a rough average of one mass extinction pulse per 110 Myr across the Phanerozoic period, following the ‘Cambrian (biological) explosion’ [2]. By this measure, mass extinctions represent a rare phenomenon in the history of life. These major reductions in the biological richness of the planet have been triggered by natural cataclysmic phenomena. For example, the combined effect of global warming and oxygen loss driven by major volcanic activity that took place towards the end of the Permian period triggered the largest mass extinction in Earth's history—the Great Dying—some 252 Myr ago [3]. Similarly, the collision of the Chicxulub meteorite on what is now the Yucatan Peninsula of Mexico annihilated much of the predominant animal life of the planet, famously the dinosaurs (save for the ancestral lineage of the birds), approximately 65 Myr ago [4].

This has been a story of biological catastrophe and recovery, characterized by a long process of biological resurgence. This delay of biological revival has been shown to be complex and dynamic, and varies depending on a plethora of factors, including, for example, the evolutionary lineage, geography and the particular mass extinction. However, a common denominator of recovery is a delay of several million years [5–8]—an important lesson for humanity.

On the other hand, a post-extinction tree of life evolves in a new configuration and becomes reconstructed with different terminal branches. For example, the truncated tree of animal life that remained after the Chicxulub meteorite crash became radically reconfigured. The diverse and often gigantic reptiles of the Mesozoic were replaced by a lineage of small-sized animals, the early mammals—a bestiary of dwarfed species that underwent a trajectory of expansion, diversification and colonization of all parts of the biosphere [9]. Another salient aspect of that bio-recovery is that over the last 100 Myr of planetary life, but particularly after the last mass extinction, a seemingly relentless trajectory of biological diversification (particularly on land) has occurred, with a dramatic, exponential buildup, leading over the 550 Myr of the Phanerozoic to a recent biodiversity pinnacle [10]. From an anthropocentric perspective, and relevant to our discussion here, three lessons stand out: (i) Earth's biological diversity does recover from mass extinction events, but this is a process that involves millions of years; (ii) the identity of the organisms that rise from the ashes, and the configuration of the communities and ecosystems they become part of, are very different from those of the ‘normal’ period before the extinction event; and (iii) we, as a species, have evolved just at a time in which the diversity of living companions is the highest in the entire history of life.

Occurring after a shorter inter-extinction interval than the Phanerozoic average—indeed at about 60% of that average interval—there is now a clear signal of the start of the sixth mass extinction episode [11,12]. Furthermore, the cause of the sixth mass extinction is a very different type of cataclysm: expansion of one element of biodiversity to planetary dominance. In short, that is, expansion of the human enterprise—the explosion of the numbers of *Homo sapiens* and their domesticates and the near-instantaneous (in terms of geological time) burst of ecosystem altering and destroying technologies. That expansion has created a new geological epoch, dubbed the Anthropocene [13,14]. The term Anthropocene, meant to replace the formal, geologically accepted label of the Holocene epoch, encapsulates the consequences of humanity's activities on Earth's life-support systems. Indeed, humanity's planetary impact includes alterations of geological processes so profound as to leave stratigraphic signatures in multiple structures of the Earth's surface. These new structures are technofossils like plastics, metal junk, radioactive wastes and other synthetic material footprints [15]. Therefore, the term Anthropocene is increasingly penetrating the lexicon of not only the academic socio-sphere, but also society more generally (e.g. it is now an entry in the *Oxford English Dictionary*) and is useful for discussion of the sixth mass extinction.

The Anthropocene includes a plethora of manifestations in terms of the activities of humanity and the translation of those into a variety of environmental repercussions [16]. The former are represented by a set of socio-economic variables underlying *growth*, including, for example, the growth rate of the number of vehicles, of fertilizer consumption, of water consumption, of the number of dams, of fast-food mega-businesses, or total world gross domestic product (GDP) (see magnitudes of change in [16]). The accelerated growth rate of these socio-economic variables is driven by an explosive human population growth, particularly in countries or regions of countries where the consumption of resources is inordinately wasteful. That is, civilization is living now under a syndrome of *too many people*, with those in *‘*developed’ parts of the world consuming an unfair share of Earth's resources and all together using an unsustainable fraction of our planet's natural capital (defined by the Convention on Biological Diversity as the world's stocks of natural assets which include geology, soil, air, water and all living things; https://www.cbd.int/business/projects/natcap.shtml). Humanity in many parts of the world is overconsuming, writing cheques heedlessly on our biological resource banks and disregarding the declining balance of natural capital rather than living more frugally on its ‘interest'.

The environmental translation of these socio-economic drivers includes the correspondingly accelerated rate of greenhouse emissions (carbon dioxide, methane and nitrous oxide), leading to climate disruption on land and in oceans. Such climatic disruption encompasses global warming, with dramatic snow- and ice-thawing from polar and high-elevation lands and consequent global sea-level rise, ocean acidification and increased frequency and intensity of extreme weather events (see [14,17]). Despite the active disinformation efforts of deniers (often sponsored by big corporations and special interest groups, in particular the fossil fuel industry; see https://www.climaterealityproject.org/blog/climate-denial-machine-how-fossil-fuel-industry-blocks-climate-action), climate change-related calamities do manage to make their way into mainstream media, including movies and documentaries. This is understandable, given the increasingly vivid, short-term catastrophic consequences on humans (and their infrastructures) around the world. However, this is not the only global environmental change of the Anthropocene. Land-use change, over-drafting of soils and groundwater, rampant terrestrial and aquatic toxification, the proliferation of invasive organisms (plants, animals and pathogenic microbes) and, especially, the intimately connected loss of biodiversity are also grave manifestations of the Anthropocene [14,18].

Here we are concerned with the latter, which is one of the most critical manifestations of the Anthropocene. Save for the ethically and ecologically unsound arguments of de-extinction advocates (see [19]), the loss of biological diversity is irreversible on a time scale of interest to humanity. The loss of biodiversity could ultimately become the most pervasive global environmental change our species will face, since all taxa that have disappeared from Earth will be gone forever. Biodiversity loss is both a cause and a consequence of global environmental change. Therefore, our destruction of the global biological richness on which we utterly depend represents an unprecedented threat to the existence of civilization that could even threaten the persistence of humanity.

## 2. The drivers of biodiversity loss: an underappreciated network of synergies

Most conservation research focuses on the impact of each of the drivers of global change on biodiversity. It is critical, however, to appreciate that the overall impact is the result of the drivers interacting in multiple and complex ways, including synergies, feedbacks and nonlinear direct and indirect effects [20]. This means that analyses of individual drivers are limited in realism and conceal the multiplicity of complex causalities of biodiversity loss. For example, we can document the local loss of animal biodiversity as a result of the combined effects of overexploitation and land-use change. In our research in rainforests in Veracruz, Mexico, deforestation and fragmentation singly reduce the amount of suitable habitat needed to maintain viable populations of large animals (an indirect effect), therefore leading to wildlife declines and eventual loss of the local populations of large vertebrates [21]. However, such deforestation and fragmentation also facilitate overexploitation (a direct effect) via the access of poachers to sectors of the habitat that previously were inaccessible—a synergy that drives the local extinction of medium-sized and large mammals (in turn affecting multiple interactions between wildlife and plants). Similarly, climate warming that impacts the health of cold-adapted animals is exacerbated by the invasion of pathogens into those climatic regimes [22], creating a synergy of wildlife loss in cold environments. These examples illustrate synergies between pairs of drivers, but interactions between three or more drivers also occur. For instance, recent research has shown that animal overexploitation and habitat loss interact with climate change, leading to a reduction of frugivorous animals around the world [23]. Similarly, climate change is allowing killer whales to move north and influencing the habitats and behaviour of white whales (beluga), which are hunted by both the orcas and climate-influenced Indigenous hunters [24].

Appreciation of the complex interplay of drivers of biodiversity loss warrants future research, and it is encouraging that recent work has started to analyse the impact of combined drivers of biodiversity loss [25]. Nevertheless, the available evidence makes it abundantly clear that the impact of humanity results from a network of proximate interacting drivers that collectively represent a planetary forcing causing a major pulse of contemporary biodiversity annihilation [12,20].

## 3. Indicators of the current biodiversity crisis

Recent local, regional and global studies present diverse indications of the current biodiversity crisis. From a plant life perspective, for example, 70% of the Earth's land surface potentially occupied by plants has been altered [26]. Consistent with the onset of agriculture some 11 000 years ago, the biomass of terrestrial vegetation has been reduced by *ca* 50% [27], with an estimated loss of approximately 20% of its original biodiversity [28]. Related to this, 40% of plants have been catalogued as endangered [29]. From a zoocentric perspective, a clear pulse of Anthropocene defaunation (*sensu* [30,31]) has been demonstrated. Vertebrate biomass consisted of some 300 million tons 11 000 years ago, of which a tiny fraction corresponded to a human population of approximately 4 million [32]. By 2015, total vertebrate biomass exploded to a dramatic 1850 million tons, but this was largely composed of domesticated animals, which monopolized 76% of the total, followed by humans at 23% (7.3 billion humans by then), while wildlife was reduced to a mere 1% (not considering seals, sea lions, amphibians and birds in this study). Despite this biological holocaust, a little fewer than 700 vertebrate species have been recorded as extinct or extinct in the wild over the last 520 years [11,12]. Undoubtedly the extinction of many more species, particularly of small-sized, understudied invertebrates, has gone unrecorded [33], but a basic point remains—the holocaust is the loss of populations and the ecosystem services they provide, *not* the loss of species, as we will discuss later [34].

## 4. The extinction crisis: an intersection of two complex adaptive systems

Within an ecosystem, the plants, animals, fungi, bacteria and many other types of microorganisms play ecological roles via their evolutionary and ecological interaction with their abiotic and biotic environments. Such interactions define the functioning of ecosystems. They are complex adaptive systems, as they consist of myriad elements that interact locally (survive and reproduce), leading to emergent system properties [35]. Predicting the exact trajectory of a complex adaptive system is near impossible but predicting one that will have emergent properties is generally correct. Changing the atmospheric temperature will certainly change the functioning of a terrestrial ecosystem, but just how is much more difficult to predict.

Although of very recent appearance in the evolutionary tree, and with a few traits that set them apart, human beings are part of biodiversity and elements in a global ecosystem. Their most distinctive traits among vertebrates are their vast stores of non-genetic information or ‘culture’ [36] and their ultrasociality—levels of cooperation vastly greater than those seen in other mammals [37].

Human culture is another complex adaptive system with emergent properties (religions, wars and pandemics), but again the trajectory of the entire system is notoriously unpredictable. Combine two complex adaptive systems, and you can see why mitigating or even reversing the anthropogenic effects of the ongoing sixth mass extinction event in detail is particularly difficult (see [38]). Traffic jams are one emergent property of the cultural complex adaptive systems, but the basic problem cannot be solved by arresting drivers who slow down.

Civilization, and even the fate of our species, is utterly dependent on proper global ecosystem functioning. Ecosystem functioning, including primary productivity, the biogeochemical cycles, and the network of trophic mutualistic and antagonistic species interactions that compose the food chains, is the fabric of life—a fabric that is translated by humans as ecosystem services (e.g. [28,39]).

The vast literature on the biodiversity–ecosystem function relationship and the significance thereof in terms of services to humanity has focused its attention on the consequences of changes in the diversity of (mostly) plant species or genetic variants on four major types of ecological processes: (i) provisioning, such as crop yield, fodder yield, wood production, medicines and medicine models; (ii) regulating, such as biocontrol, pollination and nutrient cycling; (iii) support services such as primary productivity; and (iv) cultural services, such as inspiration, and education (see a classic review in [40]; also [39]). Biodiversity–ecosystem function studies focused on animals are more limited, but some reviews make such relationship evident too, including services such as crop pollination and pest control, seed dispersal, litter decomposition, carbon cycling, carrion and dung removal, soil erosion control, animal forage provisioning, and zoonosis risk regulation (see reviews in [30,41]). What all this implies, in practical terms, is that the millions of years of plant and phytoplankton cumulative photosynthesis; the tens of millions of soil organisms that transform dirt into fertile soil, decompose the bodies of dead organisms and contribute to nutrient recycling; the wild and domesticated plants, animals (both terrestrial and aquatic) and fungi that for millennia have fed and currently feed the human population (i.e. we all eat biodiversity); the communities of animals that maintain plant reproduction and genetic diversity, as well as those animals that regulate the abundance of disease hosts and vectors; the thousands of plants, fungi, other microorganisms and animals that have provided and continue to provide medicine or medicine models; the physical protection due to ecosystem ‘structures' such as mangroves and coral reefs from extreme weather events; and the increasingly appreciated significance of the inspirational, educational and emotional benefit derived from our contact with biodiversity constitute the life-support systems for humanity (see a recent review in [18]).

In a different perspective, ecosystem services have been examined in economic terms (see a major review in [42]), and several researchers have attempted to calculate the value of nature's services in a variety of ways. Among these would be the cost of infrastructure that needs to be developed to substitute for the services of, for example, protective coastal ecosystems, and the price of water treatment plants that can play the role of wetlands in filtering contaminants [43]. Similarly, one estimate is that without mangroves flood damage in tropical coastal areas would increase by more than 16% or $US82 billion annually. However, we emphasize that the fundamental value of ecosystems in the intersection culture–ecosystem functioning lies in that the value of our life-supporting systems ‘is essentially incalculable’ [18].

This short review makes it evident that humanity cannot survive in the absence of biodiversity and ecosystem functioning, which, as we have discussed above, we are increasingly degrading. Furthermore, the prospect of *Homo sapiens* being present when the normal recovery times following a mass extinction occur is simply unrealistic. Finally, it is imperative to appreciate that all these aspects of human dependence on biodiversity—the intersection between human culture and ecosystem services—occur at the level of the populations of the myriad species and functional groups present where human populations are present. Therefore, it is crucial that we examine the impact of the human enterprise on the myriad populations of plants, animals, fungi and microorganisms.

## 5. Population declines and extinctions: the heart of the impending mass extinction

We re-emphasize that the magnitude of the current extinction crisis is underestimated owing to three key factors. First, the lack of attention given to this existential threat [38]. Second, most people, even many scientists, assume incorrectly that the problem is primarily one of the disappearance of species when it is in fact the existential threat of myriad population extinctions [44]. Third, while concerned scientists know there are many individual and collective steps that must be taken to slow the rate of population extinctions, only some advocate one fundamental, necessary and ‘simple’ cure. That, of course, is reducing the scale of the human enterprise [38].

**Figure 1.**
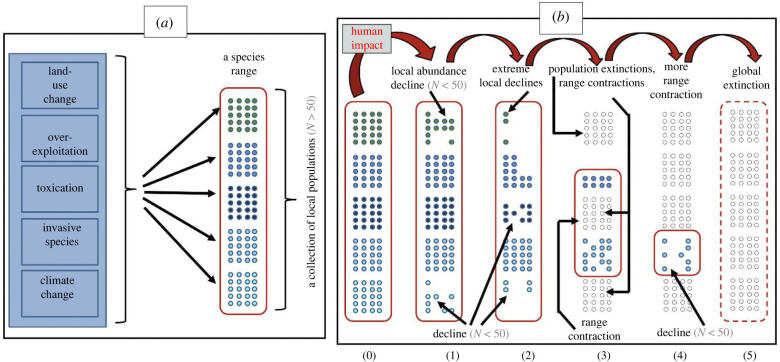
The spatio-temporal dynamics of population extinctions leading to range contraction and ultimately global species extinction. (*a*) This depicts how species are composed of mosaics of multiple locally viable populations, here considered with a hypothetical local abundance of *N* > 50 individuals (circle clusters in different shades of blue) along their distribution range, and the proximate drivers of impact. (*b*) This illustrates six stages of the extinction dynamics, starting with an unimpacted population mosaic (0), which undergoes human impact leading to local declines in abundance in some populations (stages 1 and 2), subsequently undergoing local population extinctions and range shrinkage (stage 3). Subsequent population extinctions and range contraction (stage 4) eventually lead to global species extinction (stage 5).

Let us consider, first the global extinction of species—the total disappearance of different kinds of organisms from the face of the Earth; that is the facet of the sixth mass extinction event that captures most of the attention, among both the scientific community and the public. The strong emphasis placed on numbers of extinct species leads to the misinterpretation that biodiversity is not immediately threatened but is just part of a slow episode of extinction. For example, the number of vertebrate species recorded as extinct since year 1500 is 338, or 667 if we count species extinct in the wild and those regarded as threatened (according to the International Union for the Conservation of Nature (IUCN) Species Red List). These are seemingly low numbers, in contrast with estimates of many millions of species extant. However, they result from close to 60 and 70%, respectively, occurring over just the last 120 years [11,12]. This exemplifies the ‘Anthropocene acceleration’ discussed earlier and the latter numbers represent extinction rates 100–1000 faster (depending on the vertebrate group) than the background extinction rates for vertebrates [11,12]. Recent model trajectories of bird species across IUCN's categories of endangerment concluded that the ‘effective’ bird extinction rate is six times higher than that observed since 1500 [11,12], indicating that extinction analyses should consider not only the extinct species but also the endangerment trajectory of species that are deemed not at risk now. Such a process of endangerment follows a spatio-temporal dynamic as illustrated in figure 1 (see also [45]).

Most species are constituted of a mosaic of populations distributed throughout their geographical range (figure 1*a*). Depending on the environmental heterogeneity that occurs through the range, populations of the species can be phenotypically or genetically differentiated into locally adapted populations (represented by the different shades of colour in figure 1*a*). In their native range, the individuals that make up such populations are sufficiently abundant that the populations are demographically and genetically viable (stage 0 in figure 1*b*). It is these population mosaics that are being impacted by the different drivers of anthropogenic impact—individually and in complex synergies among all these. Under such stresses, the abundance of individuals begins to decline (figure 1*b*, stage 1), with some populations reducing their densities to levels below population viability (figure 1*b*, stage 2), in some cases with populations experiencing extreme declines, leading to local population extinctions and range contractions (figure 1*b*, stage 3). As this process progresses and population extinctions continue, the range shrinks even further (figure 1*b*, stage 4), to the point that only a few populations, comprising a few individuals (therefore demographically and genetically non-viable), remain. At this stage, the species can still be counted as not extinct, even though it has experienced the collapse of its populations and humanity has lost the ecosystem services it once supplied. The extinction dynamics depicted here represent the prelude of the global extinction of species and are exemplified by numerous species of plants and animals. For example, from a sample of 177 species of mammals, just shy of 50% exhibited a range contraction of at least 80% in the period of 1990–2015 [44]. Similarly, billions of populations of plants and animals have been lost in the last centuries, and the most recent Living Planet Report indicates that the abundance of individuals of a large number of monitored species of animals has declined by 70% over the last four decades [46]. These examples constitute a vivid representation of the population extinction crisis.

Furthermore, from the point of view of the species' ecological roles within their natural communities and ecosystems, it is their local populations that really matter. Consider, for example, the case of the elephant (*Loxodonta africana*), common hippopotamus (*Hippopotamus amphibius*) and black rhino (*Diceros bicornis*), which have been exterminated in many areas of their original distribution ranges throughout Africa and South Asia [47]. This massacre means that many populations of each species have been lost (a veritable, major pulse of *within-species* biological extinction); that the ecology of the savannah (in terms of the dynamics of fire, for example) in those localities is now disrupted [47]; and that it represents a tragedy for the local populations of humans who, for example, had or might have had an ecotourism business as a way of living. All these losses occur, even though the species itself is not extinct, as it still exists somewhere else in a deplorable remnant of its former geographic range. This pattern (and the implications thereof) is consistent with that of other emblematic species, such as: the orang-utang (*Pongo* spp.), Asian rhinos (*Rhinoceros* spp.) and the Oriental pied hornbill (*Anthracoceros albirostris*); the koala (*Phascolarctos cinereus*) [48] in Australia; the jaguar (*Panthera onca*), harpy eagle (*Harpia harpyja*) [49] and tapirs (*Tapirus* spp.) [50] in Latin America; and the bison (*Bison bison*), wolf (*Canis lupus*) and grizzly bear (*Ursus arctos*) in North America [47,51].

## 6. Actions that can be taken to slow the rate of population extinctions

A number of proximate actions can be taken to prevent populations from circling the extinction drain, including the following.

### (a) Telling it like it is

Although the magnitude of the crisis is formidable, as we have outlined here, effective communication of what is at stake is central [38]. Grasping of the scale of the problem needs to go beyond the scientific arena and reach out to policy-makers and society in general. It is notable that, while climate change has drawn the spotlight, the biodiversity crisis has comparatively received appallingly little attention [38]. The young, in particular, if properly informed, can represent an ambassador with potential to help mobilize society, just as we have seen in the case of Greta Thunberg in the climate crisis. The critical grasping of the problem needs to consider that climate change and biodiversity loss are inextricably connected and, in conjunction with the other drivers of change, represent a formidable but poorly appreciated threat to humanity.

### (b) Safeguarding what is still present

Although the damage to biodiversity is considerable, we still have a few relatively unscathed remnants in the natural protected areas of the world and, to some degree, in some human-dominated landscapes. Since a large portion of such remnants of biodiversity is present in Indigenous and rural territories, recognizing, supporting and materially compensating those populations is a matter of utmost importance. In addition, safeguarding those Indigenous territories is critical to retain the traditional ecological knowledge and languages that are being profoundly eroded from these communities across the world [52,53]. This is compatible with recent efforts such as the Half Earth, championed by E. O. Wilson [54], and the 30 by 30 initiatives [55]. Safeguarding remnants of biodiversity can in turn serve as an inoculum for the agenda of restoration in the areas where this is needed and feasible. In this regard, restoration needs to go beyond traditional reforestation and consider refaunation and, ideally, the restoration of ecological processes, consequently leading to the protection or restoration of ecosystem services [56].

### (c) Moving towards an ecologically friendly human diet

The dramatic deforestation resulting from land conversion for agriculture and meat production could be reduced via adopting a diet that reduces meat consumption. Less meat can translate not only into less heat, but also more space for biodiversity and betterment of human health [57]. Although among many Indigenous populations, meat consumption represents a cultural tradition and a source of protein, it is the massive planetary monopoly of industrial meat production that needs to be curbed [41]. Related to this, the overexploitation of animals and animal products is another action that can be addressed without incurring any impact on society; on the contrary, it has the potential to reduce the perverse business of wildlife trafficking, and fresh markets that in addition represent a latent risk of zoonosis, like the one that has impacted humanity over the last two years.

### (d) Combat kakistocracy

To the extent that we engage in telling it like it is, and society becomes increasingly better informed of the risks of a ghastly future [38], we can aspire to have a societal force ready to elect leaders committed to address the biodiversity crisis and other existential threats.

## 7. An ultimately simple cure: reduce the scale of the human enterprise

It is clear that only a giant change in human culture can significantly limit the extinction crisis. Humanity must face the need to reduce birth rates further, especially among the overconsuming wealthy and middle classes. In addition, a reduction of wasteful consumption will be necessary, accompanied by a transition away from environmentally malign technological choices such as private automobiles, plastic everything, and treating billionaires to space tourism. Otherwise growthmania will win; the human enterprise will not undergo the needed shrinkage, but will continue to expand, destroying most of biodiversity and further wrecking the life-support systems of humanity until global civilization collapses [38]. Avoiding that, with its vast increase in death and misery, will require simultaneous increases in equity—not just gender equity to increase fairness and discourage over-reproduction, but equity in general so that people can be assured they are not being asked to shoulder more than a fair share of the substantial burdens the transition to sustainability will entail. Dealing with the emergent properties of the two interacting complex adaptive systems we have described here would be difficult enough without conflict further complexifying both [35].

Circling the drain is dizzying even for scientists documenting it. All people well enough off to pay attention to issues beyond their immediate needs and those of their loved ones are faced with arrays of serious issues buried in the cultural complex adaptive systems. Health, finances, politics, status and such, demand our attention. However, from childhood, the formal education system and the communications of civil society (MAHB.Stanford.edu) must challenge us to pay attention to the biospheric complex adaptive systems as well. The price of not doing so will end the dizziness—we will go down the environmental and cultural drain.

## Data Availability

This article has no additional data.
